# Then and Now

**Published:** 1920-10-23

**Authors:** 


### THEN AND NOW.

Pressure on our available space prevented us from referring in our last issue to the singularly interesting Presidential Address which was delivered by Sir William Hale White before the Medical Society of London and recently published.* There is always something singularly fascinating in a carefully considered retrospect, and in the case of a recently so progressive science as medicine, we see a double value in this type of essay in that far more than mere historical information may be derived therefrom. There is nothing that teaches so much to the present as the reasons for failures in the past, and the painstaking efforts made by our medical predecessors may serve in a thousand ways as an example to that prevalent type, the experimental " hustlers " of the twentieth century.

The speaker wisely selected a brief period of twenty-five years upon which to concentrate his analysis, and started at the year 1773, when the Society was actually founded. In passing, it is a point to note that the Guy's Physical Society was in fact started two years before this date—a circumstance not generally known to the men who owe their training to that hospital. But in any case the founding of such societies was a clear indication that the doctors of those days were in great earnest to add to the profession's store of practical knowledge, and various abstracts of the old " proceedings " which Sir William quoted show very clearly with what an excellent and well-balanced discrimination between casual conjunction and necessary connection the problems of the time were discussed. At least these men could truly appreciate the value of evidence.

Comparisons are neither invariably pleasant nor directly helpful, but are we sure that as much could be said of some of the rushed debates of to-day? In the old times their deliberations were prolonged—or should we say exhaustive? One on fermentation alone lasted four consecutive evenings; one on animal vitality is credited with six. Cases were recorded with an exactitude and detail that might well be imitated to-day. These old memoirs conclusively prove that our forefathers were, above all, highly trained observers. They had relatively few instrumental aids, and correspondingly had need to make use of their simple senses to a degree which is to-day chiefly remarkable for its absence as far as the great average is concerned. Although they made what we now know to have been mistakes, there is little doubt that we are to-day making errors just as great.

If we seek, in defence of this statement, the then greatest source of error, we find it to lie apparently far more in therapeutic than in diagnostic work. It is indeed quaint to realise, for example, that although the value of ipecacuanha for dysentery was widely known, the major actions of digitalis appreciated, and many of the standard drugs of to-day quite successfully and intelligently applied, yet all this was occurring side by side with no little attention paid to the phases of the moon in relation to therapeutics!

The use of quinine, or rather of Peruvian bark, was extraordinarily popular for all manners of ills and might be indirectly accounted for, as Sir William explained, by the well-known fact that malaria was then rife in many parts of Britain, although it had not then been differentiated from a great variety of other conditions. In a percentage of cases its administrations would naturally produce a dramatically beneficial result and we may well forgive its trial in not a few febrile states which were, unfortunately, not malarial. From whichever particular aspect of Sir William Hale White's address the reader derives his greatest interest, there is no doubt that great lesson which it carries concerns the urgent need that working among a great crowd of accessory means towards diagnosis of disease we should not for one moment forget the paramount value of simple power of accurate, unaided observation. The profession is indebted to him not only for the pains he has taken to abstract some of the most illustrative records of the past, but also for the clarity with which he brought their meaning to the general workers of to-day.

* The *Lancet*, October 16.

